# Genomic basis for early-life mortality in sharpsnout seabream

**DOI:** 10.1038/s41598-022-21597-3

**Published:** 2022-10-14

**Authors:** Héctor Torrado, Cinta Pegueroles, Nuria Raventos, Carlos Carreras, Enrique Macpherson, Marta Pascual

**Affiliations:** 1 Centre d’Estudis Avançats de Blanes (CEAB-CSIC), Car. Acc. Cala St. Francesc 14, 17300 Blanes, Girona Spain; 2 Department of Genetics, Microbiology and Statistics, and Institute for Research on Biodiversity (IRBio), University of Barcelona, Av. Diagonal 643, 08028 Barcelona, Spain; 3 Otolith Research Lab, Centre d’Estudis Avançats de Blanes (LEOV-CEAB-CSIC), Car. Acc. Cala St. Francesc 14, 17300 Blanes, Girona Spain; 4 Present Address: Island Evolution Lab, Marine Laboratory, University of Guam, 303 University Drive, 96923 Mangilao, Guam USA

**Keywords:** Population genetics, Genetic association study, Evolutionary ecology

## Abstract

Mortality at early life stages of fishes is common in nature and can be shaped by stochastic and selective processes. Selective mortality has rarely been assessed in natural conditions but can now be studied by combining genomic data with information on different life stages that realates to fitness. Here we investigate selective mortality between settlers and six-month survivors of the sharpsnout seabream by genotype-phenotype/environmental association studies in three localities along a geographic gradient. We gathered information on 105 individuals at 85,031 SNPs, obtained from individual based 2b-RAD libraries, as well as 9 phenotypic and environmental variables derived from individual otolith readings. We found common signals across localities for potential selection, such as lower survival rates for individuals hatching earlier, growing faster and experiencing higher temperatures during their planktonic phase. We identified 122 loci with parallel significant association to phenotypic and environmental variables. Importantly, one of these loci mapped to the exonic region of the *il20rb*, a gene involved in immune response, in the phylogenetically closest reference genome, showing parallel frequency changes in non-synonymous mutations in the three studied populations. Further temporal assessments are needed to understand how polymorphisms that are key to selective mortality are maintained.

## Introduction

One of the main topics of fish ecology is to understand the causes determining the number of juveniles recruiting to the adult population each year^1^. This year-class strength is regulated by the number of eggs and larvae produced by the reproductive stock, the offspring arriving from the planktonic phase to the benthic population (settlers), as well as by post-settlement mortality^2^. The mortality during this post-settlement period is usually size-selective, with small settlers having a lower survivorship than larger ones^3,4^. Some larval characteristics, such as the length of the pelagic larval duration (PLD), the pelagic larval growth rate (GRPLD), the size at hatch or the size at settlement can affect this size-selective mortality rate, modulating the year-class strength in numerous species^3,5,6^. Moonlight during the lunar cycle can also affect survival, differentially influencing both adults and offspring^7^. On the other hand, some studies have demonstrated the existence of genetic differences between recently settled larvae and survivor juveniles^5,8^. Previous studies have reported a negative relationship between allelic richness and mortality rate^9^, changes in allele frequencies among age groups^10^, or the association of certain alleles with juvenile growth rate and survival^11^. To date, addressing the genetic basis of survival at early-life stages, its association to phenotypic and environmental variables, and understanding its consequences at the population level is an open question for many species.

High-throughput sequencing technologies and specifically restriction enzyme-based reduce genome representation tools (RAD-seq) have revolutionized the field of population genetics. These techniques provide a cost-effective approach to population genomics in non-model species, since they can be used in the absence of a reference genome^12–20^. Among these techniques, 2b-RAD produces tags of uniform length, using type IIB restriction enzymes, which cleave genomic DNA upstream and downstream of the target site^21^, and obtain good results even for degraded DNA samples^16^.

In most fish, individual-based information on early life history traits can be obtained from otoliths (bones in the inner part of the ear)^22^ and used to obtain individually-based environmental information^23^. This data has been successfully combined with genomic data to infer fine-scale dispersal^24^ and for genome-wide phenotype and environmental association studies^17^. Thus, the study of otolith-inferred individual larval traits, combined with genomic data can allow for the evaluation of the genetic basis of traits associated with survival that exhibit the same trends across multiple locations.

**Figure 1:**
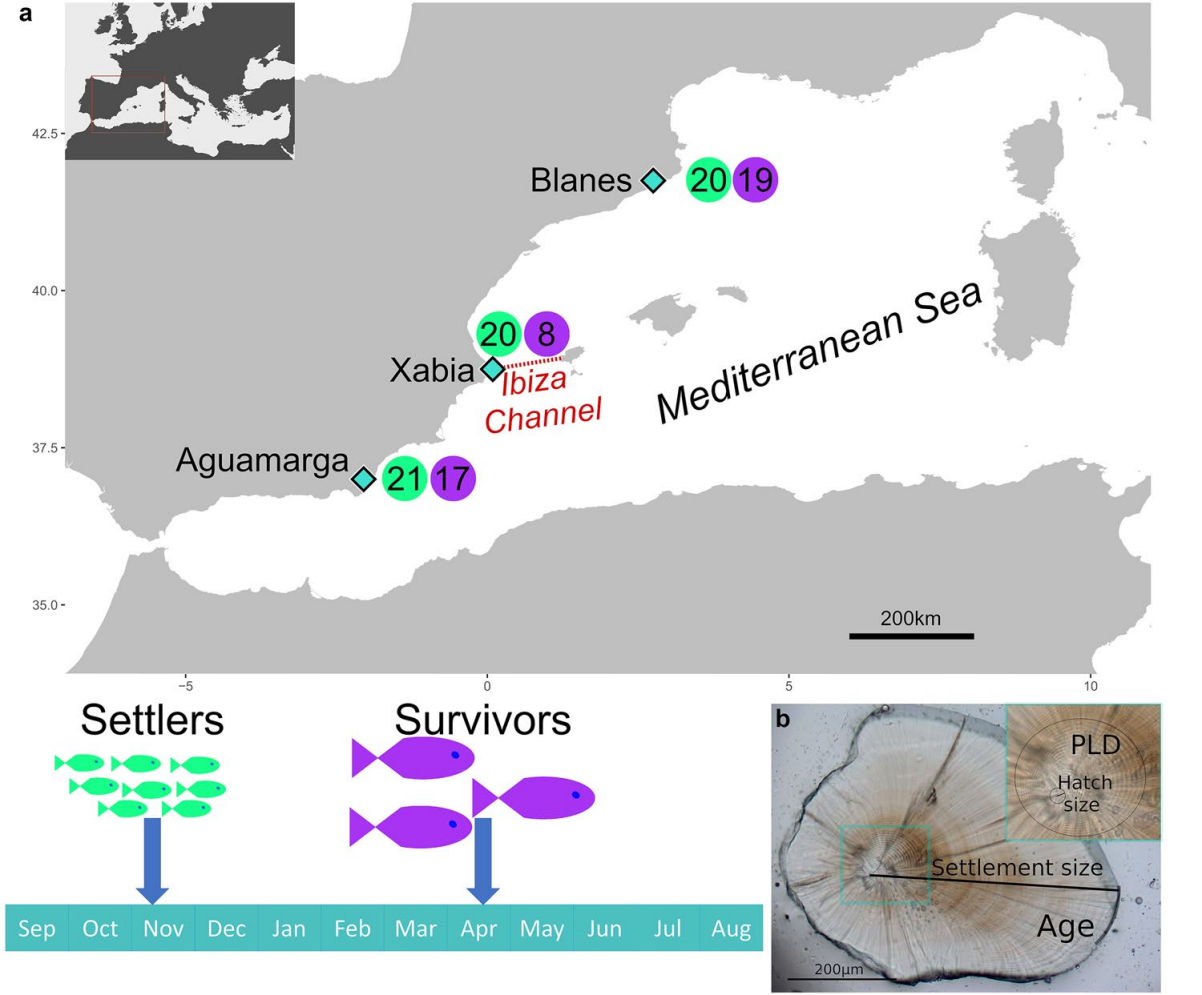
Sampling sites and experimental design to test for selective mortality in *Diplodus puntazzo*. (**a**) Location of the three sites (Blanes, Xabia and Aguamarga) where recently settled individuals (in green) were collected in November 2015 (Settlers) and individuals surviving winter (in purple) were collected in April 2016 (Survivors). The number of individuals analysed in each age group and site are given in the circles. Map was made using R ggplot2 package^53^. (**b**) The otolith of each individual was used to obtain individual hatch, larval and settlement phenotypic variables (hatch date, hatch size, settlement date and settlement size, pelagic larval duration (PLD) and pelagic larval growth rate) and environmental variables (moon phase at hatching and settlement, and temperature during pelagic larval phase).

In this work, we aim to elucidate the genomic basis for early-life selective mortality using the sharpsnout seabream, *Diplodus puntazzo* (Walbaum, 1792), as a model system. This fish is a protandrous hermaphrodite, inhabiting rocky shore reefs and seagrass meadows along the Mediterranean Sea and the Eastern Atlantic. It has an important ecological role in coastal areas, as it is the only Mediterranean fish with a wide prey spectrum, including toxic prey such as sponges, echinoderms and coelenterates. For this study, we sampled two different juvenile age groups (Fig. 1): settlers (individuals within a few days of settling from pelagic phase) and survivors (individuals surviving winter and within 6 months after settlement). Importantly, we collected these individuals in three localities in the Western Mediterranean along a geographic gradient (Fig. 1). From otolith readings, we obtained larval traits and environmental parameters. Information on nine phenotypic and environmental variables were individually obtained: three at hatching (hatch date, hatch size and moon phase at hatch), three as larvae (pelagic larval duration, pelagic larval growth rate and temperature during larval duration) and three at settlement (settlement date, settlement size and moon phase at settlement). We constructed and sequenced individual libraries using the 2b-RAD technique^16^*,* and quantified genetic and phenotypic differences between the two juvenile age groups. We evaluated differential mortality occurring at early settlement stages related to pre-larval, larval and post-larval variables. We identified candidate loci associated with selective mortality, their genomic location on a closely related reference genome, and potential functional changes of key loci. Our study reveals parallel genomic changes across populations associated with phenotypic and environmental variables. We provide a prototype for future genomic and ecological studies to evaluate the causes determining early-life selective mortality in the wild.

## Results and discussion

### Phenotypic and environmental variables associated to juvenile survival

**Figure 2:**
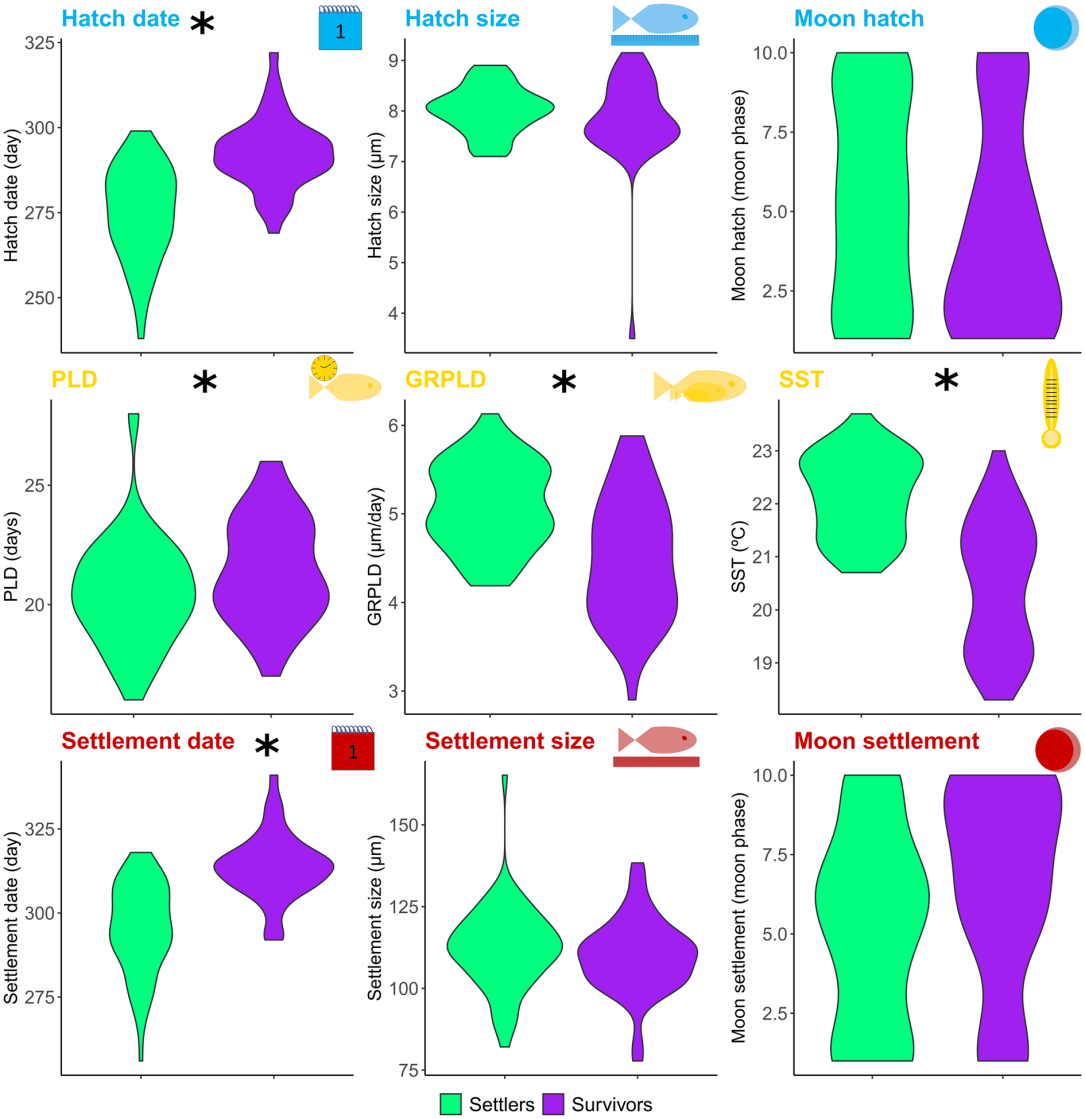
Biological and environmental signatures in early life stages in fish. Violin plots of the phenotypic and environmental values in settlers and survivors obtained from individual otolith readings during different early-life stages grouped by hatch, larval and settlement variables. Asterisks indicate significant differences between settlers and survivors according to the Permanova test, suggesting differential survival for these traits.

We first evaluated the phenotypic and environmental differences between the two juvenile age groups: settlers and 6-month survivors (Fig. 2). The PERMANOVA analysis revealed significant differences between the two age groups for hatch date, pelagic larval duration (PLD), the pelagic larval growth rate (GRPLD), sea surface temperature (SST) and settlement date (Supplementary Table S1). SST is the environmental variable explaining most of the differences between settlers and survivors (r^2^ = 0.42). Violin plots in Fig. 2 also show that both hatch and settlement dates as well as PLD values were higher in survivors than settlers, while GRPLD and SST were lower. This indicates that survivors are born later in colder waters, grow slower, and live longer as larvae.

It is worth noting that we also found significant differences among localities for all traits but settlement size and moon hatch, and a significant effect of the interaction between localities and age groups in hatch date, growth rate, SST and settlement date (Supplementary Table S1). Due to the significant effect of the locality, we analysed differences among settlers and survivors in each locality separately (Supplementary Table S2). We found significant differences in all localities for hatch date, SST and settlement date. In agreement with the findings combining all localities, survivors were born and settled later in colder waters in all cases (Supplementary Fig. S1). For GRPLD, survivors grow significantly slower in all localities except Xabia. For PLD, we found significantly higher values in survivors only in Aguamarga (Supplementary Table S2, Fig. S1). These results confirm the global findings, supporting the parallel trend of differential survival in all analyzed populations of *D. puntazzo*, in which those fish that are born later in colder waters, grow slow and live longer as larvae, and are more likely to survive. Predation patterns may influence the abundance of survivors^25^. Settlers of *D. puntazzo* are preyed upon by many different predators, including cnidarians, cephalopods and fishes, suffering a clear density-dependent mortality during the first month after the peak of settlement^26^. Settlers arriving later usually form smaller and less dense shoals than individuals arriving earlier^27^, and could suffer a lower mortality rate as it has been observed in other fishes^25,28^.

### Genomic differentiation across juvenile age groups and localities

We obtained genomic information from the 105 sampled *D. puntazzo* individuals, using the 2b-RAD technique, and sequenced a mean of 7.7E6 ± 1.5E6 reads per individual. After filtering, we retained 85,031 SNP loci corresponding to 58,095 haplotype loci, with a mean depth per locus of 29.67 ± 13.54 (see Methods section for details).

Within populations, there is a lack of genetic differentiation between settlers and survivors according to the *F*_ST_ analysis, but we detected significant differentiation in settlers between Blanes (northernmost) and Xabia and Aguamarga, with no difference between the two latter localities (Supplementary Table S3). This structuring seems logical as it divides the localities by their position with respect to the Ibiza Channel (Fig. 1), a well-known oceanographic front located in the area^29^. In particular, Blanes is situated north of the channel, while the other two sites are south. Previous studies reported the effect of this front in genomic structuring in other pelagic fish species such as *Serranus cabrilla*^30^ and *Symphodus ocellatus*^17^. In agreement with our findings, oceanographic larval dispersion models for *D. puntazzo* have shown little exchange between localities located on either side of the front, despite some larvae ultimately passing through^23^. However, in survivors, genetic differentiation was smaller and only significant between Blanes and Xabia.

**Figure 3:**
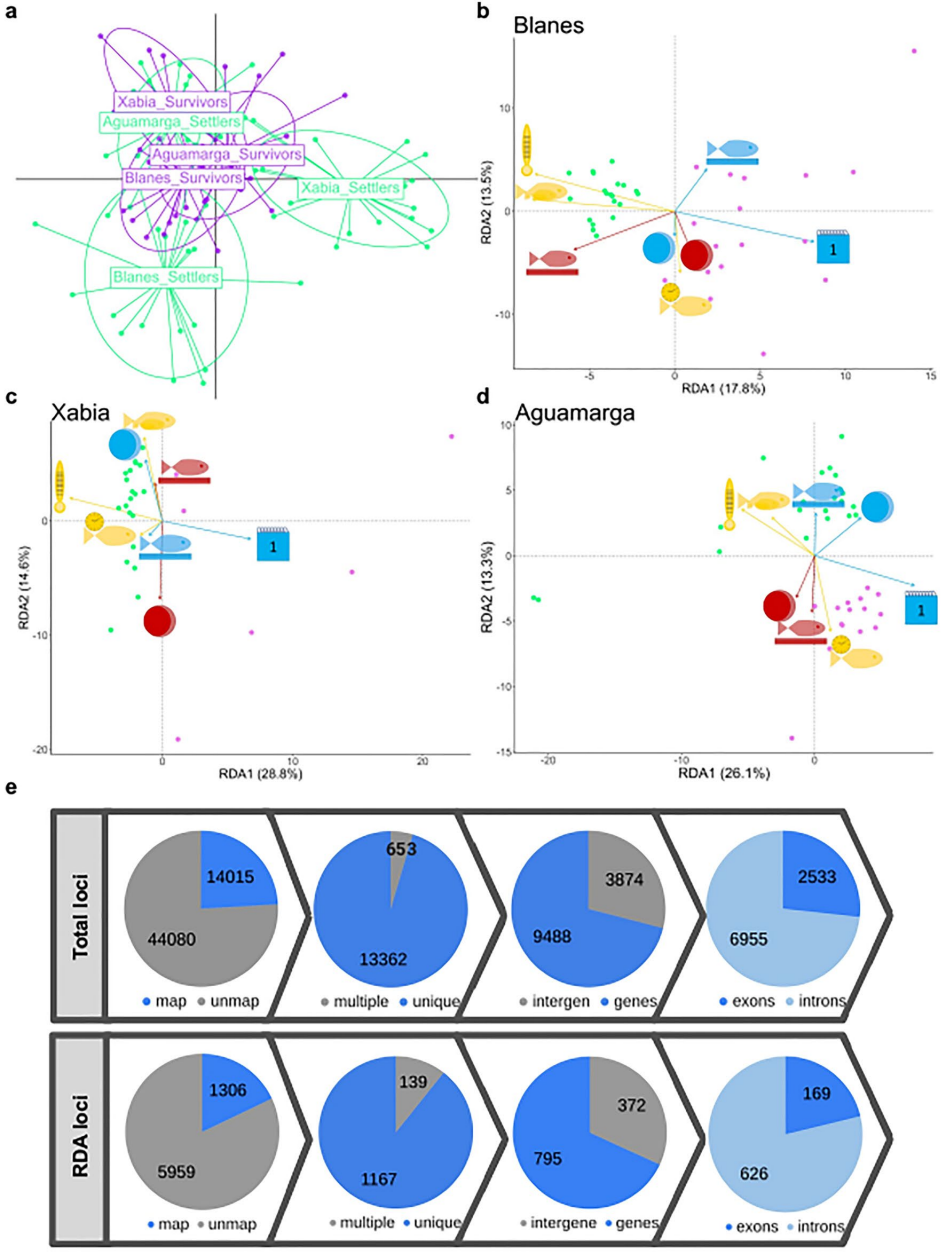
Genomic structure of settlers and survivors and structural distribution of candidate loci associated with phenotypic and environmental variables. (**a**) Discriminant Analysis of Principal Components (DAPC) plot of settlers (green) and survivors (violet) of the three localities based on 58,095 haplotype loci. (**b–d**) Redundancy Analyses (RDA) performed with individually based genotypic, phenotypic and environmental data for each locality based on 85,031 SNPs. Vectors indicate phenotypic and environmental predictors of the two first RDA components, green dots correspond to settlers and violet dots to survivors. Predictor symbols are the same as in Fig. 2. (**b**) Blanes, (**c**) Xabia and (**d**) Aguamarga. (**e**) The distribution of all analysed loci of *Diplodus puntazzo* loci according to blast searches in the annotated genome of *Sparus aurata* (top) and the RDA candidate loci (bottom): from left to right number and frequency of mapped and unmapped loci, unique and multiple loci, loci in genic and intergenic regions, and loci in exons and introns.

In agreement with the above results, the Discriminant Analysis of Principal Components (DAPC) analysis also shows genetic differentiation among localities, especially among settlers (Fig. 3a), indicating that settlers are more genetically differentiated than survivors. This suggests that survivors keep only a small fraction of their initial genetic variability. Genetic differences among age groups have been described in other species. For example, allelic selection was found in *Neopomacentrus filamentosu*s and *Diplodus sargus* after 3 and 4 months of monitoring settlers using allozymes and mtDNA haplotypes respectively^11^. An influence of size-selective predatory exposure on genetic composition was found in *Dascyllus aruanus* based on microsatellite loci^31^. Taken together, this suggests that the genetic composition plays an important role in selective mortality in early phases of fish. By combining all our results, we see that the genetic composition slightly changes from settlers to survivors, and that settlers were responsible for a greater extent of the differentiation observed between populations. If we consider that survivors are a small subsample of settlers, these differences in genetic composition may be explained by a bottleneck-like situation, as only a tiny proportion of settlers survives, coupled with selective mortality, in which specific genotypes are selected in these early life stages.

### Phenotypic and environmental associated SNPs for juvenile survival and functional characterization

We first evaluated the extent to which the studied phenotypic and environmental variables are correlated, and we discarded Settlement date from further analysis since it showed a correlation with Hatch date higher than 0.8. The Redundancy Analysis (RDA) to identify the genomic variability associated with phenotypic and environmental factors with parallel responses in each locality, clearly separated individuals by age group (Fig. 3b–d), supporting the idea that settlers and survivors are differentiated in all sites. Our results show that Hatch date exerts directional pressure affecting all the populations studied. However, previous studies suggested that this variable can vary according to different environmental factors among species or populations^17,32–34^. GRPLD is also known to affect pre- and post-settlement phases but, again, the direction of selection depends on the species. While some studies show a lower growth rate for the survivors, as we find for *D. puntazzo*^35–37^, other studies have found the opposite in different species^38–40^. Temperature is a well-known factor that can act as a selective pressure for many marine species. Nevertheless, its effects are sometimes complex to see and interpret, as it may affect species in different ways, such as changes in metabolism and larval survival and development, among others^17,41,42^. In our case, a density-dependent mortality has already been observed in *Diplodus puntazzo*, *D. sargus* and *D. vulgaris* in our area of study^26^. This mortality can act as a selective pressure for the hatching date and, in the long term, could cause a shift in the spawning dates over the years, as seen in *Theragra chalcogramma*^32^. As *D. puntazzo* reproduces from August to October^42^, this shift to later Hatch and Settlement dates would result in development in colder waters, which would explain the lower GRPLD, as it is reduced in lower temperatures^42^.

The RDA analysis allowed us to identify 7265 loci significantly correlated with phenotypic and environmental predictors. These candidate loci included 9035 SNPs, most of them (81.7%) associated with a single population (Supplementary Fig. S2, Table S4). We evaluated whether the candidate loci were enriched in a particular genomic location, i.e. genic (distinguishing between exons and introns) and intergenic. To do so, we located all *D. puntazzo* loci in the *S. aurata* genome by performing Nucleotide BLAST searches (Fig. 3e, Supplementary Table S4). The percentage of mapping was significantly lower for the RDA associated loci than for the total loci (chi-square = 87.764, p-value < 10E^05^), indicating that a larger fraction of the RDA associated loci are located in low-conserved regions not shared with *S. aurata*. Importantly, the majority of loci (> 89%) mapped to a unique location in the *S. aurata* genome, which is a requirement to unambiguously identify loci. 71% of loci that mapped to unique regions were found within genes and, most of them (73%) were located in intronic regions. The RDA subset was significantly enriched in intronic regions compared to all loci (chi-square = 11.202, p-value = 0.0008).

We also investigated the functionality between data sets by comparing gene biotypes and performing a functional enrichment analysis on the associated GO terms (see material and Methods for details). We did not detect significant differences when comparing gene biotypes of the uniquely mapped loci (chi-squared: 3.347, p-value is 0.34; Supplementary Table S4). The RDA loci are related to a plethora of functions, reinforcing the idea of complex mechanisms involved in early-life stage survival (Supplementary Fig. S3). The functional enrichment analysis indicates that the functional distribution of the protein coding genes associated with the RDA and the total loci are not significantly different.

Altogether, most RDA loci are located in regions that cannot be evaluated with the current data available and would require mapping to a phylogenetically closer reference genome. We hypothesize that most of the RDA associated loci are located in fast evolving regions that may be involved in regulatory functions or *D. puntazzo* specific genes. Nonetheless, our results show that the low fraction of RDA mapped loci behave similarly to the total loci. Future studies using the genome of this species (when available) will provide a more complete picture of the genetic mechanisms involved in differential survival.

### Parallel genotype-phenotype/environmental association of early-life survival across localities

**Figure 4:**
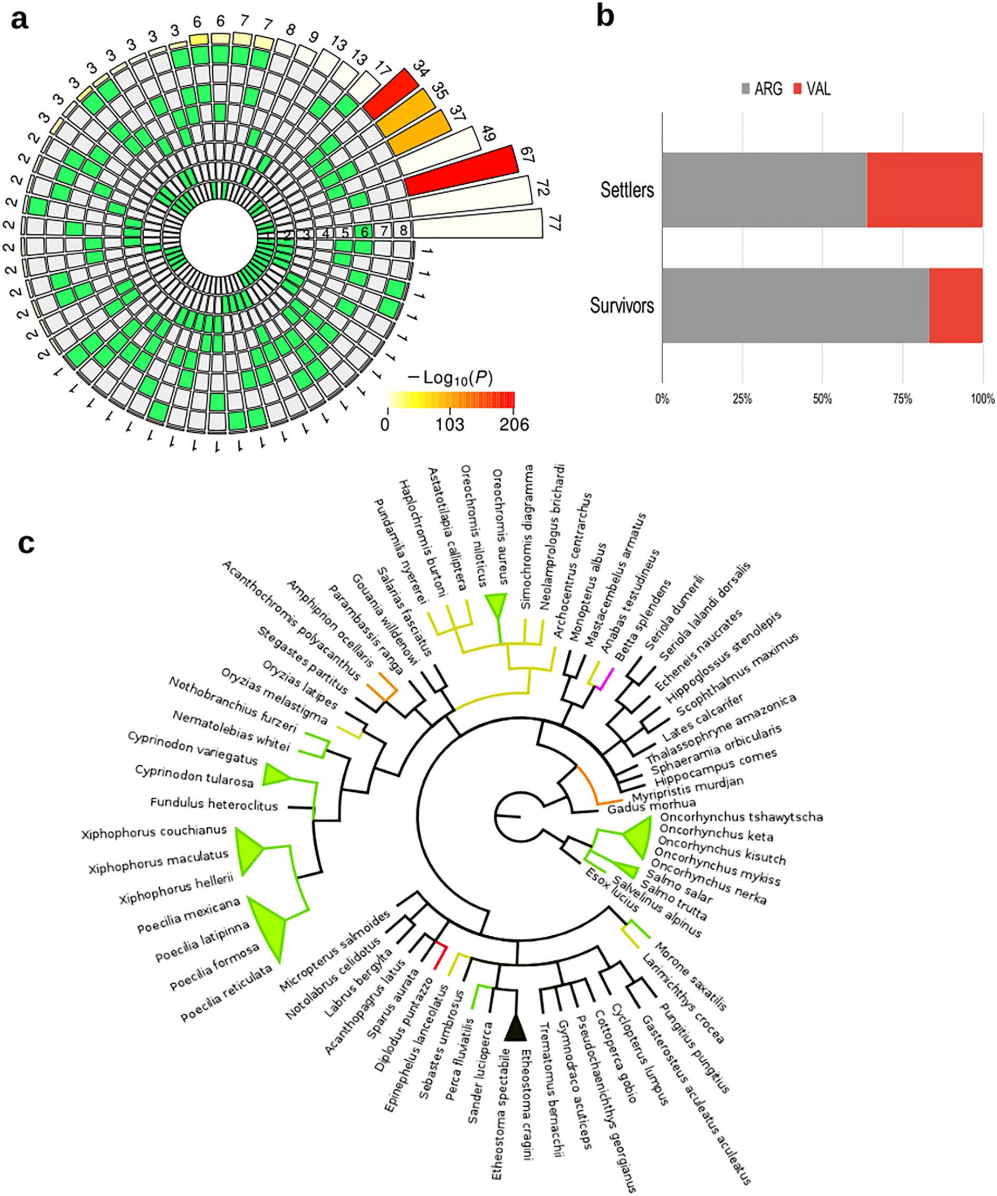
Environmental and phenotypic associations of the parallel set of loci. (**a**) Categorization of the loci with significant associations to phenotypic and environmental variables in the three populations. The variables are designated with numbers: 1: Hatch date, 2: Hatch size, 3: Moon hatch, 4: PLD, 5: GRPLD, 6: SST, 7: Settlement size, 8: Moon settlement. The eight inner circles indicate in green the presence of a significant association between each variable and the identified SNPs. The colour of the outer circle represents the P-value significance of the category, constituted by one or multiple associated variables, and the number of SNPs found in that category is given on top of each bar. (**b**) global amino-acid frequency in the three populations for settlers and survivors for the polymorphism found in the parallel locus mapping to the coding region of the Interleukin 20 Receptor Subunit Beta (Locus_94259) and associated with hatch date, SST and moon set. (**c**) neighbour-joining tree of 74 fish species. Labels are coloured according to the amino acid found in position 197, corresponding to the polymorphic allele represented in (**b**).

We identified 122 RDA loci containing 133 SNPs significantly associated with select phenotypic and environmental variables and having the same tendency in the three populations (i.e., being positively or negatively correlated to the same variable), when comparing settlers and survivors. These loci are strong candidates to understand the genetic basis of adaptation to the studied predictors and hereafter are referred to as parallel set loci as they show parallel responses in all populations. The parallel set is significantly depleted in mapped loci compared to the total (chi-square = 4.602, p-value = 0.03) since only 14% of them mapped to the *S. aurata* genome. Interestingly, 81% of the parallel set loci were localized in genic regions and all corresponded to protein coding genes (Supplementary Table S4). We checked which phenotypic and environmental predictors are interacting with the parallel set of loci (Fig. 4a). SST, GRPLD and hatch date are the predictors showing significant associations with a higher number of SNPs, ranging from 34 to 67 depending on the group of overlapping variables. Moon set is also significantly interacting with SST, hatch date and GRLPD and those interactions are found in a minimum of six loci.

Three loci from the parallel set mapped to exonic regions (Locus_53477, Locus_93132 and Locus_94259). However, the first two did not show allele frequency changes between settlers and survivors (Supplementary Table S5) and, therefore, they are not expected to have a central role in the differential survival detected. On the contrary, Locus_94259 showed significant differences in allele frequencies when comparing settlers and survivors (Fig. 4b, Supplementary Table S5). This locus is associated with hatch date, SST and moon set, and maps to the coding region of the Interleukin 20 Receptor Subunit Beta (il20rb). This gene affects the immune response mediated by cytokine receptor and interleukin-20 binding activities. The two SNPs detected in *D. puntazzo* are located in the first and second codon positions, resulting in an amino-acidic replacement from arginine (major allele) to valine (minor allele) in position 197 of the *il20rb* gene (XP_030259501.1) from *S. aurata* (R197V). Importantly, survivors have a significantly lower proportion of the valine allele than settlers (Fig. 4b, Supplementary Table S5), suggesting that this replacement may play a role in survival. To evaluate the degree of conservation of this position we compared the ortholog sequences of 74 fish species (Fig. 4c). We detected a total of six different amino acids in this position, with arginine being the most frequent (48.6%). The frequency of the other amino acids found in this position was in agreement with their physico-chemical properties and Blosum62 substitution scores, with those more similar to arginine being the most frequent (lysine: 31.1%, threonine: 13.5%, serine: 4.1%, methionine: 1.4% and valine: 1.4%, Supplementary Table S6). Thus, it seems that this position allows for some variability but is generally restricted to amino acids with similar characteristics that may not affect the functionality of the gene. We also generated a homology model of the secondary structure of the *S. aurata* gene that shows that this amino acid replacement is found in a lamina beta located in the D2 domain (Supplementary Fig S4). The model shows interactions with seven amino acids from the same chain, suggesting that this position may be key for the stability of the structure. The D2 domain is located adjacent to the membrane and seems to influence intracellular signal transduction pathways and important cellular responses^43^. Altogether, it seems that the valine replacement affects the functionality of the gene, which may explain the significant lower amount of this allele in survivors. However, there could be an antagonistic pleiotropy effect (*i.e.*, a differential beneficial effect at different life stages) which may explain the increase of the valine allele in settlers. Thus, this gene may be involved in selective survival in *D. puntazzo.* However, since the percentage of mapped loci was low, other relevant candidate loci could be missed. Overall, our study shows that the mechanisms involved in early-stage survival are likely to be complex, with the (many) loci underpinning it associated with a plethora of functions. It is worth noting that our study is limited to a single cohort. Previous studies have found unexpected patterns of spatial and temporal genetic structure where genetic variation should be homogenized^24,44^. This chaotic genetic patchiness (CGP) is common in fish with pelagic early-life stages, but has not been addressed in *D. puntazzo* specifically. Our study shows no significant differentiation within populations between age groups (Table S3) and in consequence CGP is not expected to be relevant in this species. However, further studies in consecutive years are needed to confirm this hypothesis.

## Conclusions

We find clear signals of selective mortality in settlers of sharpsnout seabream mostly associated with hatch date, sea surface temperature, and growth rate during the pelagic larval phase. The parallel genetic changes identified by the environment/phenotype-genotype associations support a genetic basis of differential mortality between settlers and survivors. While there are a large number of loci associated with these early-life traits, most of them do not map to the closest available genome, suggesting that they are located in low-conserved regions, and highlighting the necessity of having phylogenetically closer genomes. Temporal studies through space and time are needed to corroborate the patterns and processes of selective mortality mediating local adaptation.

## Methods

### Sampling and data collection

The sharpsnout seabream *(Diplodus puntazzo,* Walbaum, 1792) is a shallow-water (0–30 m) coastal fish species. It has a larval duration of 16–29 days and is a representative component of the rocky and sea-grass beds communities of the Western Mediterranean^45,46^. Its reproductive season takes place from August to October^42^. The first settlers of *D. puntazzo* usually arrive at the beginning of October or November, with settlement timing varying between localities in the NW Mediterranean Sea^47^. The settlement period is usually very short (2–3 weeks), and the peak abundance is attained just 10 to 20 days after the onset of the settlement period. No additional 1.0 to 1.5-cm specimens are observed one week after the peak, and this is taken to mark the end of the settlement period^27^. Survivors (individuals within 6 months after settlement) need to be collected before mid-May when dispersal of *D. puntazzo* juveniles to the area outside the survey grid starts, at a mean individual size of about 5 cm^27^.

We sampled 61 settlers, between the second half of November and beginning of December 2015, and 44 survivors, in April 2016, from Blanes, Xabia and Aguamarga using hand nets and stored in 96% Ethanol (Fig. 1). We extracted and mounted the otoliths of each individual on a microscope slide. Otoliths show daily increments, as well as hatch and settlement marks (Fig. 1). These traits were determined using a light microscope following the standard methodology described in the literature^4,22^. We analysed the otoliths from the centre to the edge along the longest radius, and the hatch date was estimated by subtracting the total number of daily increments from the sampling day (Fig. 1). We calculated the pelagic larval duration (PLD) as the number of daily increments between the hatch and the settlement marks, and used this value to estimate the settlement date. We obtained the size (in µm) at hatch as the radius from the centre to the hatch mark and at settlement as the radius from the centre to the settlement mark. We measured the otolith three times and used their mean to minimize measurement errors. Mean error was on average 1.75% and always less than 4.5%. We used these measurements to obtain information on nine phenotypic and environmental variables for each individual: three at hatching (hatch date, hatch size and moon phase at hatch), three as larvae (pelagic larval duration, pelagic larval growth rate and temperature during that period) and three at settlement (settlement date, settlement size and moon phase at settlement). We measured the growth rate during the pelagic larval duration as the increment in otolith size in µm divided by PLD in days as in Raventos & Macpherson^4^. Reproductive patterns and offspring survival in fish have been shown to vary across the lunar cycle^7^, and thus we have included as variables the moon phase during the hatch and settlement dates. The moon phase is expressed as a categorical variable with 10 levels each representing 10% of the period between two consecutive new moons. The temperature during the pelagic larval period (SST) was calculated as the mean daily value in the 10 km around each locality during the corresponding period for each individual. The original temperature values were obtained from the SOCIB system (Balearic Islands Coastal Observing and Forecasting System).

In order to evaluate the possible differences of early-life traits between settlers and survivors, Permutational Multivariate Analyses of Variance (PERMANOVA) were performed, one for each phenotypic and environmental variable, as implemented in the R function ‘adonis’, from the ‘vegan’ package v 2.5-2^48^. These analyses were carried out for all samples combined and for each locality independently.

### DNA extraction and genotyping

DNA was individually extracted using the QIAamp DNA Mini Kit (QIAGEN) following manufacturer’s instructions. DNA integrity was checked by gel electrophoresis and quantified by NanoDrop or Qubit. To build individual genomic libraries with 2b-RAD we followed the protocol described in Barbanti and co-authors^16^. In brief, the Genomic DNA of each sample was individually digested by *AlfI*. Adaptors with degenerate bases (5′-NN-3′) were ligated to the sticky ends of the digested sequences. Barcodes and Illumina primers were attached to the adaptors by PCR. Purification was performed using magnetic beads, quantified using PicoGreen® and samples pooled and single end sequenced (50 bp) with a HiSeq 2500 Illumina at the Center for Genomic Regulations (CRG) of Barcelona. Some of the settlers of Blanes (N = 12) and Xabia (N = 12) were sequenced previously using the same methodology^16^ and data combined with new data from the present study for additional 37 settlers and 44 survivors. Several previously sequenced samples were repeated and showed high genotyping accuracy (mean of 98.6% coincident genotypes).

Raw sequences of all individuals used in the analysis were processed simultaneously prior to genotyping, using customized scripts^16^. Those files were used for genotyping with STACKS vs 2.53 software^49^. To construct the catalog of loci (*i.e.* the list of all loci across individuals as defined by the program) two mismatches were allowed between stacks within (M = 2) and between (n = 2) individuals, and a minimal stack depth of three was required (m = 3). Individual genotypes were outputted as SNP VCF files. Additional filters were applied using VCFtools vs 1.12^50^. Individual genotypes with a depth below 5X were not considered. Loci with a missingness value higher than 30%, and loci with a minimum allele frequency (MAF) of 0.05 were removed from the dataset with VCFtools. We also removed loci with high mean depth across all individuals, corresponding to a sequencing depth above 1.5 times the interquartile range from the dataset. Finally, the loci in HW disequilibrium at two of the three localities were discarded to remove possible paralogous loci.

### Population genomics and genotype-environment/phenotype associations of early-life mortality

We used the haplotype loci corresponding to the filtered SNPs for our population genomics analysis. F_ST_ pairwise values and their significance (computed by 999 permutations) were obtained by R software ‘hierfstat’ package vs 0.04-2^51^. A Discriminant Analysis of Principal Components (DAPC) was performed retaining a number of PCAs equal to one third of the number of individuals. The analysis was performed with the R software package ‘adegenet’^52^ and represented with the ‘ggplot2’ package^53^.

We used Redundancy Analyses (RDA) to evaluate genotype-environmental/phenotypic associations for each locality independently. We followed the method described by Forester and co-authors^54^, identifying as outliers those loci with more than three standard deviations from mean loading (equivalent to a two-tailed p-value = 0.0027). We used a genotypic matrix with all the SNPs in all loci as the response variable and as predictors the eight phenotypic and environmental variables, after discarding the settlement date, which was highly correlated to the hatch date. We finally identified the SNPs significantly associated with the three studied populations having the same directional genomic response to the same phenotypic/environmental variable in all populations, hereafter referred to as parallel SNPs.

To identify if candidate loci are enriched for certain genomic regions (i.e., genic, intergenic, exonic, intronic), we conducted BLAST searches with the 34 bp sequences of all loci against the genome of *Sparus aurata* Linnaeus, 1758. We selected *S. aurata* since it is the phylogenetically closest available reference genome to *D. puntazzo*, as reported in a previous study on the Sparidae family^55^. To do so, we performed local Nucleotide BLAST searches (Blast v2.10.1) against the *S. aurata* genome downloaded from NCBI (GCA_900880675.1), using an E-value cut-off of 10^–4^. We then classified blast hits as mapped to a unique location or to multiple locations. The fraction of uniquely mapped reads was further classified as exonic, intronic and intergenic using an in-house python script (https://github.com/EvolutionaryGenetics-UB-CEAB/classifyBlastOut.py).

We classified the loci that mapped to genic locations according to the gene biotype of the mapped gene (protein coding, long non-coding RNA, pseudogene, tRNA). In addition, we assigned GO terms to genic loci that mapped to protein coding genes using the following pipeline: we selected the *S. aurata* genes with blast hits mapped (either exonic or intronic), and we extracted the longest protein encoded for each of them [5252 protein coding *S. aurata* genes (Supplementary Table S4)]. We then functionally annotated the selected proteins by assigning GO terms using eggNOG-mapper v5.0^56^ on the EMBL server (http://eggnog-mapper.embl.de/, accessed October 2021). To do so, we did two types of analyses, we prioritized *bona fide* assignments by limiting to one-to-one orthologs within Chordata (TaxID 7711) and, we maximized coverage by considering many-to-many orthologs within Chordata. We then matched *D. puntazzo*, *S. aurata* and GO terms using an in-house python script. Finally, we performed a functional enrichment analysis of the RDA *loci* versus all mapped loci using FatiGO^57^. Revigo was used to reduce and visualize GO terms^58^.

### Characterization of the candidate loci

We first quantified the number of intersections between the phenotypic and environmental predictors and the parallel set of loci using the SuperExactTest package from R^59^. We calculated whether the proportion of alleles found in settlers and survivors are significantly different using a Chi-squared test. We identified the genes mapped in the Nucleotide BLAST searches (see above) and checked their functionality using Uniprot (www.uniprot.org). We identified orthologs of the *il20rb* gene from 74 fish species using Protein BLAST searches to RefSeq genomes (https://blast.ncbi.nlm.nih.gov/, accessed October 2021). We randomly selected one gene for each species and we obtained multiple alignments and a Neighbor-joining tree using Mafft with default parameters (https://mafft.cbrc.jp/alignment/server/, accessed October 2021). The physico-chemical properties of the amino acids and the Blossum62 substitutions scores for Arginine were checked on https://www.ncbi.nlm.nih.gov/Class/Structure/aa/aa_explorer.cgi and https://microbenotes.com/amino-acids-properties-structure-classification-and-functions/#properties-of-amino-acids. We built a homology model of the *il20rb* gene using the crystallized protein of the human ortholog Q9NYY1 (4DOH) using SWISS-MODEL (https://swissmodel.expasy.org/interactive, accessed October 2021).

### Ethical compliance

The collection of fish samples was conducted in strict accordance with Spanish and European regulations. The study was found exempt from ethics approval by the ethics commission of the University of Barcelona since, according to article 3.1 of the European Union directive (2010/63/UE) from the 22/9/2010, no approval is needed for fish sacrifice with the purpose of tissue or organ analyses. Furthermore, the study species, *Diplodus puntazzo*, is not listed in CITES. All methods were carried out in accordance with relevant guidelines and regulations.

## Supplementary Information

Supplementary Tables.

Supplementary Figures.

## Data Availability

Raw read data,genotypes and phenotypic/environmental data from each individual will be available in the European Nucleotide Archive (ENA) at EMBL-EBI under accession number PRJEB52256 (https://www.ebi.ac.uk/ena/browser/view/PRJEB52256) upon acceptance.

## Code availability

The scripts used for all analyses are archived through Github (https://github.com/EvolutionaryGenetics-UB-CEAB/).
