# Supplementary material for: Genomic basis for early-life mortality in sharpsnout seabream
Source: Sci Rep. 2022 Oct 14;12:17265. doi: 10.1038/s41598-022-21597-3 (PMC9568528; doi:10.1038/s41598-022-21597-3)
Supplement: Supplementary file 2 — Supplementary Figures. [file 41598_2022_21597_MOESM2_ESM.docx]

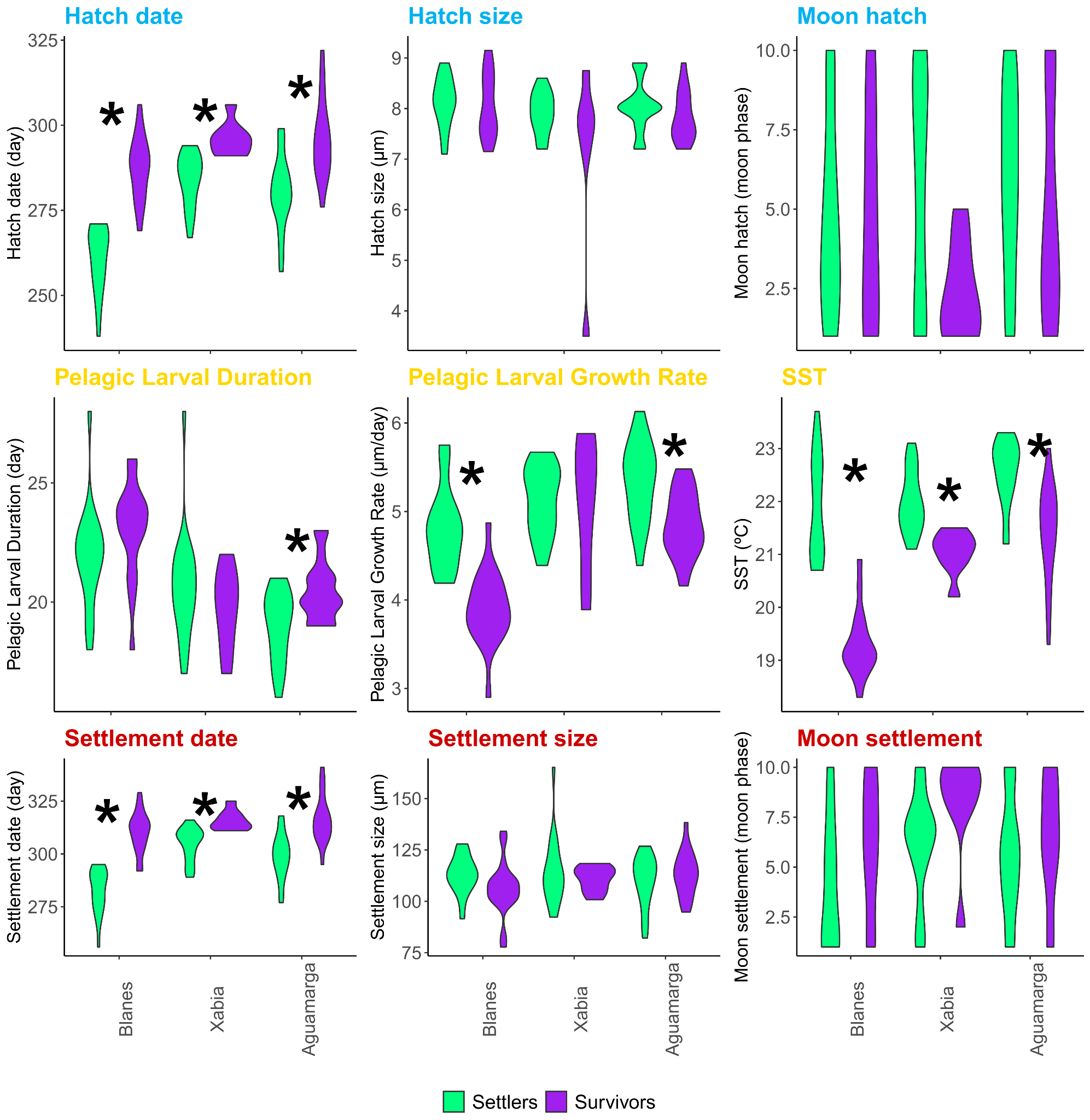


**Fig. S1:** Violin plots of the phenotypic and environmental values in settlers and survivors in the three analysed populations. Asterisks indicate significant differences between settlers and survivors according to the Permanova test, suggesting differential survival for these traits.

**
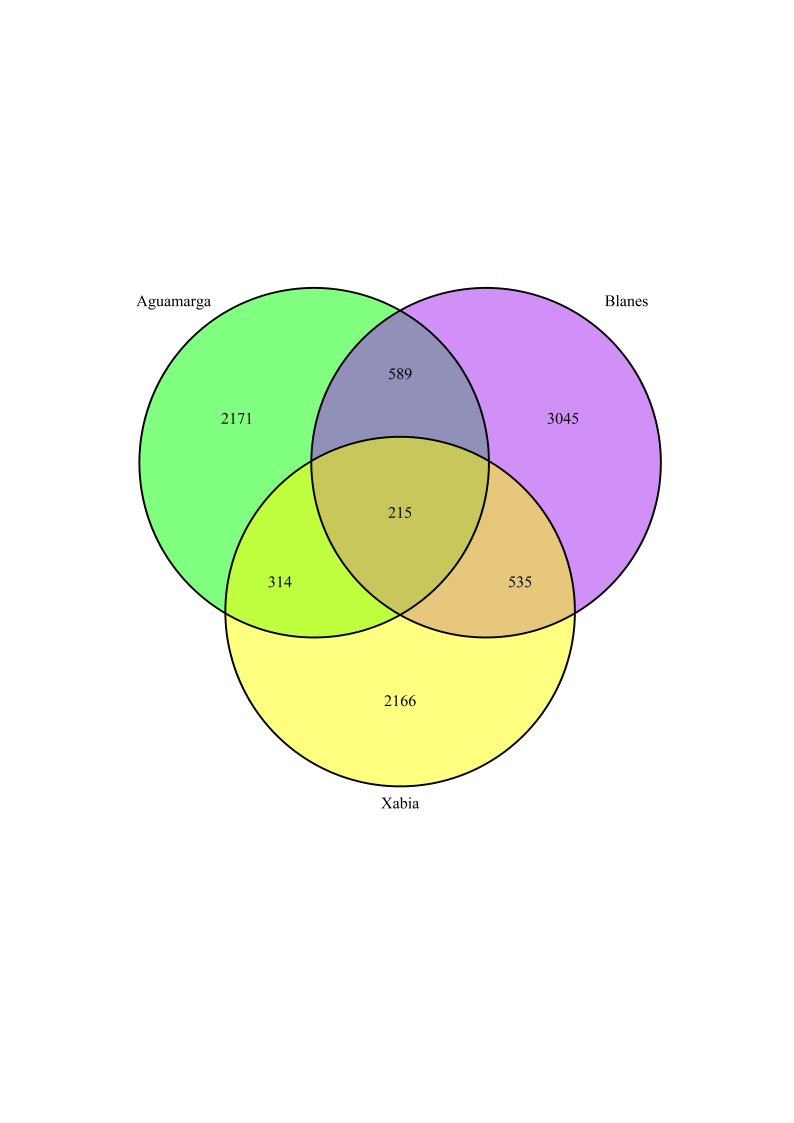
Fig. S2:** Venn diagram showing the RDA loci associated to each analysed population.

**
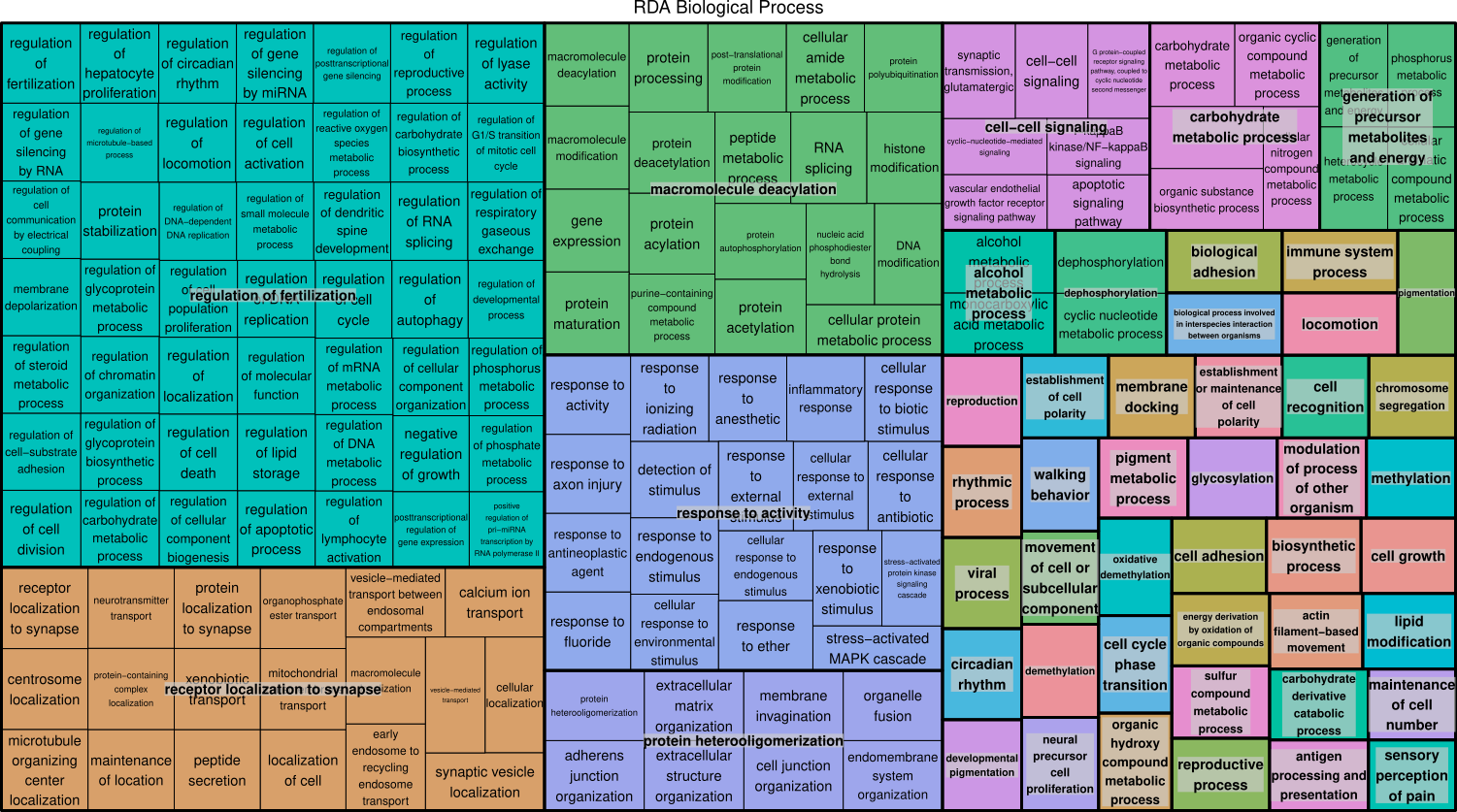
Fig. S3:** GO terms from the Biological Process category associated with more than two RDA loci. GO terms were assigned from one-to-one orthologs.

**
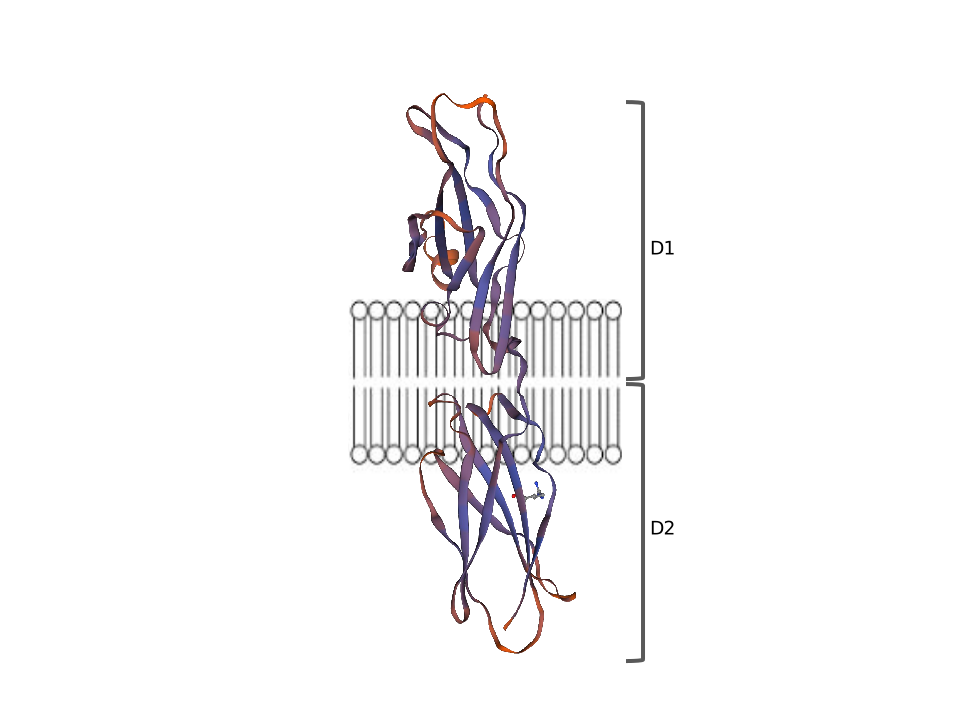
Fig. S4:** Homology model of the secondary structure. Colors designate the accuracy of the model, being blue regions the better modeled positions. In D2 there is the chemical structure of the arginine amino acid.
